# Dominance reversals: the resolution of genetic conflict and maintenance of genetic variation

**DOI:** 10.1098/rspb.2023.2816

**Published:** 2024-03-13

**Authors:** Karl Grieshop, Eddie K. H. Ho, Katja R. Kasimatis

**Affiliations:** ^1^ School of Biological Sciences, University of East Anglia, Norwich Research Park, Norwich NR4 7TJ, UK; ^2^ Department of Ecology and Evolutionary Biology, University of Toronto, Toronto, Canada M5S 1A1; ^3^ Department of Molecular Biosciences, The Wenner-Gren Institute, Stockholm University, 10691 Stockholm, Sweden; ^4^ Department of Biology, Reed College, 3203 SE Woodstock Blvd, Portland, OR 97202, USA; ^5^ Department of Biology, University of Virginia, Charlottesville, VA 22904, USA

**Keywords:** antagonistic pleiotropy, balancing selection, dominance reversal, genetic conflict, genetic trade-offs, genetic variation

## Abstract

Beneficial reversals of dominance reduce the costs of genetic trade-offs and can enable selection to maintain genetic variation for fitness. Beneficial dominance reversals are characterized by the beneficial allele for a given context (e.g. habitat, developmental stage, trait or sex) being dominant in that context but recessive where deleterious. This context dependence at least partially mitigates the fitness consequence of heterozygotes carrying one non-beneficial allele for their context and can result in balancing selection that maintains alternative alleles. Dominance reversals are theoretically plausible and are supported by mounting empirical evidence. Here, we highlight the importance of beneficial dominance reversals as a mechanism for the mitigation of genetic conflict and review the theory and empirical evidence for them. We identify some areas in need of further research and development and outline three methods that could facilitate the identification of antagonistic genetic variation (dominance ordination, allele-specific expression and allele-specific ATAC-Seq (assay for transposase-accessible chromatin with sequencing)). There is ample scope for the development of new empirical methods as well as reanalysis of existing data through the lens of dominance reversals. A greater focus on this topic will expand our understanding of the mechanisms that resolve genetic conflict and whether they maintain genetic variation.

## 1. Introduction

Explaining the maintenance of genetic variation has been a mainstay pursuit in evolutionary biology since the modern synthesis [1–9]. One mechanism of particular interest is genetic trade-offs. A genetic trade-off occurs when alternative alleles are expressed in more than one context (i.e. habitat, developmental stage, trait or sex) with differing fitness effects between contexts. Alleles that are unambiguously beneficial across contexts can be fixed by positive directional selection. Those alleles that are unambiguously deleterious can be removed from the population. However, selection can maintain genetic variation when it favours alternative alleles in alternative contexts (e.g. antagonistic pleiotropy [10–13], sexually antagonistic selection [14,15], and some forms of spatially or temporally varying selection [16–19]) or can at least prolong the loss of additive genetic variance since antagonistic alleles will have longer transit times to fixation than unconditionally beneficial/deleterious alleles [15]. There is abundant evidence consistent with antagonistic genetic variation underlying some proportion of variance in fitness or its components due to trade-offs between alternative life history traits [20–25], tissue types [26,27], sexes [28–39], environments or generations [40–44].

Until and unless the lower net-fitness allele of these antagonistic polymorphisms is lost, this form of genetic variation will impose a genetic load [2,45–48] on population mean fitness, as selection is either maintaining or slowing the loss of alleles that harm the fitness of some individuals in some context(s). Theoretical and empirical evidence suggest there is strong selection pressure to resolve this form of genetic conflict [49–51].

One mechanism that partially resolves the genetic load imposed by antagonistic genetic variation is beneficial reversals of dominance, where alternative antagonistic alleles are dominant in their beneficial context and recessive in their deleterious context [10,14,17–19,52]. For example, a dominance reversed sexually antagonistic polymorphism would entail the female-beneficial (male-detrimental) allele having dominant fitness effects in heterozygous females but recessive fitness effects in heterozygous males, and the reverse for the male-beneficial (female-detrimental) allele. Similarly, under antagonistic pleiotropy between survival and reproduction, a heterozygous individual would have the harmful effects of each allele masked by fitness benefits of the other allele, one having dominant benefits to survival but recessive harmful effects on reproduction and *vice versa* for the other allele. Hence, context-dependent dominance reversals partially resolve the genetic conflict by allowing heterozygous individuals to be closer in fitness to that of the preferred homozygous genotype of each context, improving context-specific (and hence population) mean fitness – a reduction in the genetic load. Note that even complete dominance reversals cannot completely resolve the genetic conflict, as there will always be some homozygotes in the ‘wrong’ contexts.

Whereas other forms of resolving genetic trade-offs such as gene duplication [53–56], sex-linkage [57–59] and epigenetics [60,61] may result in the loss of genetic variation, partial resolution by dominance reversal actually promotes the maintenance of the polymorphism [62]. Dominance reversal results in marginal overdominance (a net heterozygote advantage when averaged across contexts) or potentially overdominance (true heterozygote advantage) when the genetic trade-off is between individual fitness components [10], as in the Soay sheep ([24]; details in Empirical examples). Higher (mean) heterozygote fitness stabilizes the polymorphism and can deterministically maintain this form of genetic variation for fitness ([10,14,17–19,52]). The strength of selection to resolve genetic trade-offs hinges on whether the cost of the conflict (magnitude of genetic load) is great enough and therefore the degree to which they have already been (partially) resolved by competing mechanisms [53]. For example, a dominance reversal that partially resolves a genetic trade-off would reduce the strength of selection for a gene duplication at that same polymorphic locus [53]. Hence, the timescales and constraints surrounding dominance reversals relative to other forms of resolution are likely highly consequential to the fate of genome architecture, genetic load, genetic variation and the adaptive potential of the population.

Early scepticism over the plausibility of dominance reversals [12,63,64] may have delayed our understanding of this fundamental concept, but likely stemmed from the limited empirical evidence prior to the last decade [65,66]. For example, alternative variants of *Est*-4 in *Drosophila mojavensis* were shown to preferentially hydrolyze alternative esters and the heterozygotes faintly resemble the more efficient homozygote on each substrate [67] (table 1). Similarly, alternative *LDH* allozyme variants in the fish *Fundulus heteroclitus* showed opposite substrate affinity in alternative temperatures and heterozygote enzyme activity resembles the more efficient homozygote in each temperature [68] (table 1). And heterozygous amylase activity in *D*. *melanogaster* was closer to the low-activity homozygote in starch food and closer to the high-activity homozygote in normal food [69] (table 1). While these examples may seem only distantly related to organismal fitness in nature, recent empirical evidence is more convincing, putting dominance reversal back in the spotlight.

**Table 1.** Empirical evidence for beneficial reversals of dominance.

study	species	evidence	context	data	method	analysis
Zouros and Van Delden [67]	*Drosophila mojavensis*	single locus	environments	phenotypic	crossing scheme	enzyme assay
Place and Powers [68]	*Fundulus heteroclitus*	single locus	environments	phenotypic	crossing scheme	enzyme assay
Matsuo and Yamazaki [69]	*Drosophila melanogaster*	single locus	environments	phenotypic	crossing scheme	enzyme assay
Via *et al*. [70]	*Acyrthosiphon pisum*	polygenic	environments	fitness	crossing scheme	statistical modelling
Posavi *et al*. [43]	*Eurytemora affinis*	polygenic	environments	fitness component	crossing scheme	statistical modelling
Chen *et al*. [71]	*Drosophila melanogaster*	polygenic	environments	gene expression	transcriptomic	allele-specific expression
Johnston *et al*. [24]	*Ovis aries*	single locus	traits	fitness component	pedigree/GWAS*	statistical modelling
Le Poul *et al*. [72]	*Heliconius numata*	inversion / polygenic	traits	phenotypic	crossing scheme	image analysis
Gautier *et al*. [73]	*Harmonia axyridis*	single locus	traits	phenotypic	crossing scheme	image analysis
Mérot *et al*. [74]	*Coelopa frigida*	inversion	traits / sexes	fitness component	experimental evolution	statistical modelling/numerical simulations
Jardine *et al*. [75]	*Drosophila melanogaster*	single locus	traits	fitness component	genomics/phenotyping	statistical modelling
Barson *et al*. [35]	*Salmo salar*	single locus	sexes	fitness component	capture-recapture/GWAS*	statistical modelling
Grieshop and Arnqvist [36]	*Callosobruchus maculatus*	polygenic	sexes	fitness	crossing scheme	dominance ordination
Pearse *et al*. [76]	*Oncorhynchus mykiss*	inversion	sexes	fitness component	capture-recapture	statistical modelling
Geeta Arun *et al*. [77]	*Drosophila melanogaster*	polygenic	sexes	fitness component	experimental evolution	statistical modelling
Mishra *et al*. [78]	*Drosophila melanogaster*	polygenic	sexes	gene expression	transcriptomic	allele-specific expression

* = genome wide association study.

In this review, we describe the theory of dominance reversals, their ability to partially resolve genetic conflicts and their relationship to genetic variation. We then highlight empirical examples consistent with dominance reversals across several subdisciplines. Finally, we provide a forward look at identifying dominance reversals using quantitative genetics and gene expression analyses. Taken together, dominance reversals are not only plausible but supported by accumulating theory and empirical evidence, and their ability to partially resolve genetic trade-offs could render tests of dominance reversal (paired with validation tests) a powerful complement to existing methods of identifying antagonistic polymorphisms in the genome.

## 2. Evolutionary causes of dominance reversals

The plausibility of dominance reversals starts with the plausibility of antagonistic polymorphisms. A direct extension of Fisher's geometric model [79–82] shows that for relatively well-adapted populations, the relatively rare instance of a mutation that would improve fitness in one context would tend to reduce fitness in a second context (assuming similar fitness landscapes between contexts and no mutational biases) [83–85]. Hence, antagonistic polymorphisms can readily arise but will tend to be unstable if simply additive.

**Figure 1.**
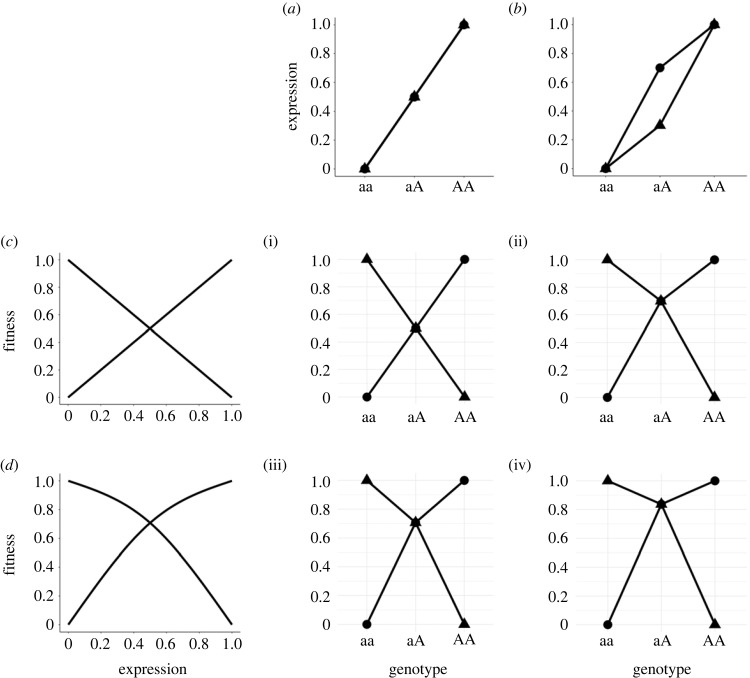
Graphical representation of beneficial dominance reversals. Circles versus triangles represent alternative sexes, environments or generations (antagonistic pleiotropy scenarios not shown). We first map genotype to trait expression under two scenarios: (*a*) additivity for the phenotype, and (*b*) dominance reversal for the phenotype. Then we map trait expression to fitness under two scenarios of antagonistic selection: (*c*) additivity for fitness and (*d*) curved fitness functions overlapping in their concave vicinities. The ultimate effect of a genotype on fitness is given by the combination of the genotype-phenotype map (*a* or *b*) and the phenotype-fitness map (*c* or *d*) in the matrix of resultant genotype-fitness panels (i–iv): (i) additivity, (ii) dominance reversal owing to the genotype-phenotype map or (iii) owing to the phenotype-fitness map, and (iv) a greater magnitude of dominance reversal owing to their combined effects. See electronic supplementary material, figure S1 for varying parameter settings, convex fitness functions, heterozygote inferiority, etc.

Whether an antagonistic polymorphism is dominance reversed, or can become dominance reversed before it is lost, is clearly important and raises the question of how dominance reversals arise. The simplest answer is that antagonistic polymorphisms may be inherently dominance reversed – an inevitable outcome of overlapping and concave fitness functions (figure 1). Theory suggests that fitness functions are generally curved in the vicinity of their optima and that beneficial alleles should be dominant [83,86–90], with modest empirical support [91–96] (but see [97–100]). Hence, for a trait with context-antagonistic effects on fitness, a de facto dominance reversal will ensue at the underlying polymorphism(s) as long as the fitness functions overlap in their concave vicinities (figure 1*d*, (iii); [17,19,88,101,102]). Still, the shape of the fitness landscape cannot be taken for granted. For example, two fitness functions could very plausibly overlap in their convex vicinities (e.g. overlapping tails of two Gaussian curves), sometimes resulting in net underdominance (heterozygote inferiority; electronic supplementary material, figure S1*e*,v,vi), which would destabilize the polymorphism and rapidly fix one or the other alleles [47]. Whether the overlap between two real fitness functions occurs in their linear (figure 1*c*), concave (figure 1*d*) or convex (electronic supplementary material, figure S1*e*) portions can only be resolved by empirical estimates of context-specific fitness functions. But dominance reversals need not rely on curved phenotype–fitness relationships since they can apparently be dominance reversed already at the phenotypic level (figure 1*b*), as in many of the empirical examples we discuss below (table 1). Note, however, that trait-level dominance reversals may or may not be dominance reversed for fitness, *per se*, depending on how the phenotype maps to fitness. In principle, dominance reversals at the phenotypic level could occur due to traits being threshold-like (non-linear genotype–phenotype relationship) [103], or could arise due to the explicit action of a dominance modifier [62].

A dominance modifier could be any genetic or epigenetic process that can affect the dominance properties between alleles at an otherwise additive polymorphism. Unlike the de facto dominance reversals owing to the shape of the fitness landscape, a dominance modifier conferring a beneficial reversal of dominance would need to evolve in concert with the antagonistic polymorphism somehow. For example, a feature of a pre-existent neutrally evolving regulatory network conducive to dominance reversed gene regulation could potentially facilitate an antagonistic mutation invading and sweeping to some intermediate equilibrium frequency, but there is currently no empirical or theoretical precedent for this. Instead, this has been approached from the starting assumption that an additive antagonistic polymorphism already exists with a sufficiently high frequency of heterozygotes. In that case, as Spencer and Priest [62] have demonstrated under sexually antagonistic selection, a dominance modifier mutation that partially resolves the genetic conflict of the additive antagonistic polymorphism is adaptive and will invade. This partial mitigation of the fitness costs paid by heterozygotes for carrying one of the deleterious alleles for their sex subsequently stabilizes the antagonistic polymorphism [62]. This theory is an extension of Otto and Bourguet's [104] theory, and the concept ultimately dates back more than 100 years, even prior to (but including) the classical Fisher-Wright debate [62,105,106].

There are two outstanding issues with dominance reversals owing to a modifier. The first is that they either have to pre-date the antagonistic mutation (as described above) or rely on a sufficiently high proportion of heterozygotes at an additive antagonistic polymorphism (since the strength of selection for a modifier is roughly proportional to the probability of finding it in a heterozygote for the antagonistic site) [62,104]. But as discussed, most additive antagonistic polymorphisms will be progressing toward fixation for one or the other allele, so there may only be a narrow window of opportunity for a mutant dominance modifier to invade before drift or selection causes the antagonistic polymorphism to be lost. The second issue is that it is unclear what real-life gene regulatory phenomena might constitute a dominance modifier of this sort, which could bear consequence on its ability to evolve before the polymorphism is lost or whether there could be pre-existent neutrally evolving conditions conducive to beneficial dominance reversal. Below, we provide a biophysically explicit proof-of-concept for what a dominance modifier could look like and analyse its evolution using forward-time individual-based population genetic simulations (box 1; electronic supplementary material S2). Our example is a starting point for a theoretical approach that would be useful in assessing both of these outstanding issues, as well as whether antagonistic selection on a phenotype can maintain genetic variation [109], whether dominance reversals compete with gene duplications [110] or other forms of resolution, and evaluating patterns in empirical data [78].

Box 1. **Dominance modifier**

An allele of a transcription factor polymorphism can be dominant by having a lower mismatch to the downstream binding site or by having greater allele-specific concentration, either of which give a greater fractional occupancy of the binding site [107]. In the three-part linear regulatory network below, the sex-limited regulatory stimulus, *D*, and Genes *A* and *B* are all unlinked, whereas the protein-coding domains of Genes *A* and *B*, *A_i_* and *B_i_*, are linked to their respective *cis*-regulatory binding sites, $\alpha_{i}$ and $\beta_{i}$. Only $\alpha_{i}$ and $\beta_{i}$ are allowed to mutate. The protein-coding domain of Gene *A* harbours a sexually antagonistic polymorphism with male- and female-benefit alleles, *A*_1_ and *A*_2_, that, respectively, decrease and increase the expression level of Gene *B*, [*B*]. Those alleles are sexually antagonistic because the standardized [*B*], φ, is under additive sexually antagonistic selection (as in figure 1*c*). (We note there is precedent for gene expression levels showing opposite sign correlations with male versus female fitness in *D. melanogaster* [108]).

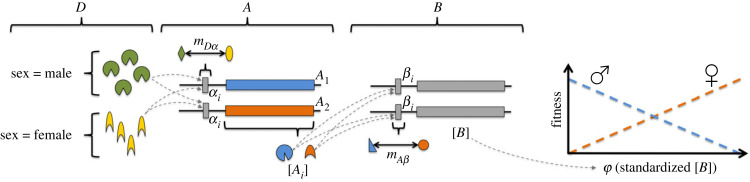

Arbitrarily, *A*_2_ is intrinsically dominant over *A*_1_ due to having a lower mismatch to the downstream substrate $\beta_{i}$ and therefore, a greater fractional occupancy of that binding site, all else equal. But *A*_1_ can be effectively dominant over *A*_2_ if it can achieve a greater fractional occupancy via increased allele-specific concentration, [*A_i_*], determined as:

B.1 $$\;[A_{i}]\;\propto \;\left(\frac{[D]/k^{m_{D\alpha }(\mathrm{sex},\;\alpha_{i})}}{1+2[D]/k^{m_{D\alpha }(\mathrm{sex},\;\alpha_{i})}}\right),$$

where [*D*] is the concentration of the sex-limited regulatory stimulus, *k* is the stepwise change in the dissociation constant for *D* binding to $\alpha_{i}$, and $m_{D\alpha }(\mathrm{sex},a_{i})$ is the proportion of mismatched nucleotides between *D* and $\alpha_{i}$ as a function of sex and $\alpha_{i}$. That is, in a heterozygous genotype $a_{1}A_{1}/a_{2}A_{2}$, mutations in *a*_1_ that reduce $m_{D\alpha }(\mathrm{male},a_{1})$ relative to $m_{D\alpha }(\mathrm{male},a_{2})$ will increase the relative concentration of the linked male-benefit allele, *A*_1_, in males.

Allowing these gene regulatory networks to evolve in forward-time individual-based population genetic simulations (electronic supplementary material S2) revealed that:

1. Selection favours the adaptive $a_{i}A_{i}$ haplotypes that enable heterozygous males and females to plastically adjust the [*B*] toward their sex-specific fitness optima (electronic supplementary material, figure S2.4) because it partially resolves the genetic conflict (electronic supplementary material, figure S2.5),
2. This dominance reversal in turn tends to maintain the focal sexually antagonistic polymorphism (electronic supplementary material, figure S2.2),
3. The ‘dominance modifier’ $\alpha_{i}$ cannot act alone and relies on the surrounding regulatory network, and
4. Our focal polymorphism exhibited a pattern of reversed allele-specific expression between the sexes (electronic supplementary material, figure S2.6) – a detectable signature of dominance reversal [78].

## 3. Evolutionary consequences of dominance reversals

Evidence of antagonistic genetic variation does not imply that the underlying polymorphisms would be maintained indefinitely under antagonistic balancing selection – most likely will not. While antagonistic mutations are inevitable (see previous section), to maintain an antagonistic polymorphism when the allelic effects on fitness are strictly additive (as in figure 1*i*) would require very strong and/or symmetric antagonistic selection between contexts [10,12,14,18,19,52,111]. However, even a partial dominance reversal would already cause a drastic expansion in the range of selection coefficients in each context that would deterministically maintain the polymorphism, including weak and/or asymmetric selection between contexts. This stabilizing effect of dominance reversal holds for genetic trade-offs between fitness components of individuals [10], between sexes [14,52] and between temporally and spatially varying environments [17–19,112] (especially in conjunction with other stabilizing mechanisms [113]).

The stability of a partially dominance reversed antagonistic polymorphism owes to the same phenomenon that renders dominance reversal a resolution to conflict: the improved mean fitness of the heterozygotes. That is, above-intermediate mean heterozygote fitness not only improves population mean fitness (resolution of conflict) but it also increases the proportion of heterozygotes, which helps both alleles stay in circulation. When the alternative contexts of a genetic trade-off are not experienced by a single individual (e.g. sexes, environments, generations) this above-intermediate fitness of heterozygotes is a net heterozygote advantage at the population level (marginal overdominance) [14,18,19], with the highest-fitness individuals still being the homozygotes in their preferred contexts (figure 1(ii–iv)). In cases where the alternative contexts are components of an individual's fitness (i.e. antagonistic pleiotropy) a dominance reversal can potentially result in true heterozygote advantage (overdominance) [10,24]. Indeed, evidence for overdominance may represent cases of dominance reversed antagonistic pleiotropy upon closer inspection, as in [24] (table 1; details in Empirical examples).

Whether overdominance or marginal overdominance, the consequence is an expansion of the range of selection coefficients that would result in stable antagonistic polymorphism in the face of random genetic drift and/or imbalances in the strength of selection between contexts [10,14,17–19,52]. This expansion of the parameter space is especially impressive for small selection coefficients [52] such as those expected on average for each of the many loci presumed to underlie fitness and continuous traits [114]. As indirect empirical support for this notion, hundreds of SNPs (single nucleotide polymorphisms) with sexually antagonistic effects on fitness in *D. melanogaster* were significantly more likely to be *trans*-species polymorphisms shared with *D. simulans* relative to a randomly selected set of frequency-matched control sites [37], meaning that most of these sexually antagonistic polymorphisms arose prior to the speciation event between the two and were maintained over approximately one million years in both species ever since, which is highly unlikely to have occurred if they were simply additive.

## 4. Empirical examples

Surprisingly, few studies have explicitly set out to test for dominance reversals. We gathered empirical evidence consistent with dominance reversals spanning several subdisciplines. In addition to the early evidence presented in the Introduction, below we highlight more recent examples of environment-specific, trait-specific, sex-specific and multi-context dominance reversals. These examples emphasize the different methodologies, forms of evidence and subdisciplines that share this common interest. Most do not directly relate dominance reversal to the resolution of genetic conflict but rather to dominance reversal assisting the maintenance of antagonistic genetic variation; however, we note that the latter is owing to the former.

### (a) Environment-specific dominance reversal

Posavi *et al*. [43] found evidence consistent with an environment-specific dominance reversal in the invasive copepod *Eurytemora affinis* (table 1). They derived two inbred strains from each of two high- and low-salinity environments and crossed them in a sex-specific full diallel cross [115] to compare survival of the within- versus between-environment F_1_ offspring in each salinity environment. They found that while the within-environment crosses showed substantially lower survival in their ‘wrong’ salinity environments, the between-environment heterozygotes exhibited high survival in both salinities. Assuming high- and low-salinity adaptation in this system in largely governed by the same loci (though it may well not be), these results are consistent with dominance reversals maintaining genetic variation under antagonistic selection between environments. This finding is like the scenario depicted in figure 1*b*, which could facilitate the maintenance of genetic variation by spatially antagonistic selection. Via *et al*. [70] identify a similar example looking at the potential for ecological speciation between two populations of pea aphid (*Acyrthosiphon pisum*) that were alternatively adapted to alfalfa and clover (table 1). F_1_ heterozygotes showed above-intermediate breeding values for fitness (per capita offspring/adult) on each host [70]. This dominance reversal is for an estimate of fitness and would likely generate marginal overdominance and balancing selection on the underlying polymorphisms [70], but the results are also consistent with heterosis (hybrid vigour) owing to divergent sets of unconditionally deleterious alleles between populations (see [102]).

Chen *et al*. [71] used allele-specific expression in two *D. melanogaster* strains and reared their F_1_ hybrids in hot or cold temperatures to identify the dominance of parental alleles under different temperature environments. They identified 1384 genes in which the opposite parental strain allele was dominant in the hot versus cold environment. However, because they assessed dominance by comparing F_1_ hybrid effects to each homozygous parent, the inferred dominance effects could be due to background genetic differences between inbred parental strains. Still, some of these candidate genes likely do represent dominance reversals for expression and this set of genes exhibit patterns that are reminiscent of our dominance modifier model (Box 1). For example, we model a transcription factor polymorphism (Box 1) and their list of 1384 genes was enriched for 13 different transcription factor binding sites [71]. Two of those 13 transcription factors were themselves putatively dominance reversed [71]. One transcription factor (*mip120*) exhibited *cis*-regulatory variation in the hot environment but *cis*- and *trans*-regulatory variation in the cold environment [71], which is consistent with our model of a dominance reversed transcription factor in that it requires the interplay between its *cis*-regulatory binding sites and a *trans*-acting regulatory stimulus (Box 1, electronic supplementary material S1). We note, however, that none of these putative dominance reversals have any known relationship to fitness or its components.

### (b) Trait-specific dominance reversal

Johnston *et al*.'s [24] study of the gene *RXFP2* in wild Soay sheep (*Ovis aries*) shows how true overdominance for fitness can emerge from dominance reversed fitness components under antagonistic pleiotropy. Homozygous *Ho^+^Ho^+^* males have relatively high reproductive success and low survival, while *Ho^P^Ho^P^* males have relatively low reproductive success and high survival. Heterozygous males have nearly equal reproductive success to *Ho^+^Ho^+^* males and nearly equal survival to *Ho^P^Ho^P^* males, which combine to yield overdominance for fitness [24] (table 1). This scenario of overdominance for fitness can occur when the genetic trade-off is between fitness components that combine to determine an individual's total fitness, even in the absence of overdominance for the fitness components ([10]). Jardine *et al*. [75] likewise identified a case of antagonistic pleiotropy between survival and reproduction likely acting to maintain short (*S*) and long (*L*) alleles of the classic *fruitless* (*fru*) gene in *D. melanogaster*. The 43 bp polymorphic indel was in a 1 kb region of the genome with elevated nucleotide diversity and Tajima's D (genomic signatures consistent with long-term balancing selection). Follow-up experiments showed that *S/S* flies had greater male mating success and lower larval survival relative to the *L* allele in its hemizygous arrangement (i.e. L/-, which we note is not the ideal comparison). Heterozygotes tended to have equally high male mating success to *S/S* flies, but also equally high larval survival to *L/-* flies (table 1). Hence, the beneficial allele for each trait was dominant for that trait's expression in heterozygotes.

Colour hierarchies can result in alternative alleles being dominant for alternative colour patches, which can be thought of as different traits. For example, Le Poul *et al*. [72] investigated the individual genes lying within a super gene that underly a mimicry polymorphism for wing colour pattern with important fitness consequences in the butterfly *Heliconius numata*. All eight within-population allele-pairs studied show opposite parental alleles being dominant for different colour patches in F_1_ offspring [72]. Perhaps the clearest case of a dominance reversal between colour patches is between the *tar* and *arc* alleles, where the *tar* allele is dominant to the *arc* allele with respect to its large black patch but recessive with respect to its white patch [72]. These dominance reversals may assist the stable maintenance of these widespread and persistent mimicry polymorphisms by limiting the production of intermediate, non-mimetic individuals [72]. In a similar case, Gautier *et al*. [73] show that harlequin ladybird beetles (*Harmonia axyridis*) with alternative *cis*-regulatory alleles of the *pannier* gene have distinct colour patterns on their elytra, and their heterozygous offspring exhibit a unique colour pattern owing to alternative alleles being dominant in some colour patches but recessive in others (table 1). This dominance reversal may contribute to the maintenance of alternative *pannier* alleles [73], as alternative colour variants seem to be subject to seasonally fluctuating natural and sexual selection [116].

### (c) Sex-specific dominance reversal

One of the turning points in the growing appreciation for dominance reversals came from Barson *et al*.'s [35] study of the *VGLL3* gene in Atlantic salmon (*Salmo salar*), which explains 39% of phenotypic variation in age at sexual maturity. Alternative early- (*E*) and late-development (*L*) alleles are under sexually antagonistic selection with males preferring the former and females the latter, but heterozygous male and female development resembles that of the *EE* and *LL* homozygous genotype, respectively [35]. While this may depend on population- and age-specific genetic architecture [117,118], it nevertheless may still partially resolve the genetic conflict between the sexes over this life history trait. In another example of a life history trait under sexually antagonistic selection, Geeta Arun *et al*. [77] revealed a polygenic signal of dominance reversal by challenging replicate populations of *D. melanogaster* to evolve in response to pathogenic infection. After 65–75 generations of experimental evolution, the progeny of crosses between populations both within and between treatments were challenged to survive infection. The between-treatment male and female progeny were not perfectly intermediate between the within-infected and within-control crosses, rather, female heterozygotes exhibited above-intermediate survivorship (closer to that of the infected group) while males showed below-intermediate survivorship [77]. The results were consistent across all four replicate pairs of the experimental evolution program, suggesting a trade-off between immunocompetence and some other fitness-related trait in males. Both studies represent examples of a sex-specific dominance reversal at the phenotypic level (as in figure 1*b*), which would likely assist in the stable maintenance of the underlying genetic variation via sexually antagonistic selection.

Grieshop & Arnqvist [36] uncovered a polygenic signal of sex-specific dominance reversal for fitness in seed beetles (*Callosobruchus maculatus*). They used a full diallel cross among 16 strains and obtained replicated estimates of sex-specific competitive lifetime reproductive success for all combinations. They then used the ‘array covariances’ [115,119] to order the strains by how dominant their fixed allelic variation was relative to one another, separately for the male and female data. They found a negative genetic correlation between the male and female dominance ordination among strains, implying that strains' fixed genetic variation tended to be dominant to that of other strains in one sex but recessive to that of other strains when measured in the opposite sex, a polygenic signal of sex-specific dominance reversal for fitness [52]. Mishra *et al*.'s [78] recent study of allele-specific expression in *D. melanogaster* also identified evidence of polygenic sex-specific dominance reversal. In 176 of 3796 quality-controlled genes, opposite alleles were significantly more highly expressed in opposite sexes (with as many as 26/176 representing false positives) [78]. This sex-specific dominance reversal for expression is consistent with a pattern predicted by our model (Box 1, electronic supplementary material, figure S1.6), but as with Chen *et al*.'s [71] study these have no known relationship to fitness or its components.

### (d) Multi-context dominance reversal

The final two studies we highlight both identified dominance reversals in major-effect autosomal inversion polymorphisms that underlie life history traits with sex-specific fitness optima. Both examples involve the interplay between life history trade-offs, environmental effects and sex- or trait-specific dominance reversals combining to consequently maintain genetic variation at these fitness-determining inversions. Pearse *et al*. [76] investigated a large supergene (*Omy05*) in rainbow trout (*Oncorhynchus mykiss*), where the ancestral and rearranged alleles represent a polymorphism that underlies an alternative migratory-based reproductive strategy subject to environmentally dependent and sexually antagonistic selection [76,120,121]. They found that the statistical model of best fit to explain their capture-recapture data was one that allowed for sex-specific dominance, which has therefore likely assisted in the maintenance of this major-effect multivariate antagonistic polymorphism for approximately 1.5 million years [76]. Similarly, Mérot *et al*. [74] examined a large inversion in the seaweed fly *Coelopa frigida*. The allelic effects of this inversion represent an environmentally dependent survival/reproduction trade-off that results in overdominance for fitness (as with other examples above), with their experimental evolution data suggesting that the overdominance emerges not only due to varying strengths and directions of dominance effects between life history traits but also between the sexes [74]. These examples highlight how multiple forms of antagonistic selection and dominance reversal can act in concert to maintain genetic variation.

## 5. Detecting signatures of dominance reversals

It is a longstanding problem to confidently detect antagonistic balancing selection using traditional methods [113,122–127] driving various creative analyses of polygenic signals [37,38,108,128–131]. For example, Ruzicka *et al*.'s [37] GWAS would have overlooked many sexually antagonistic SNPs (none being significant after Bonferroni correction) if not for reducing their significance threshold and testing for trans-species polymorphism (see end of Evolutionary consequences section, above). Their method was optimized for detecting additive signals [37], meaning that the effects would have needed to be strong enough and/or additive enough to be detected through the noise of the unmodelled dominance effects predicted of such sites. Interestingly, there is very little overlap between Ruzicka *et al*.'s [37] list of candidate sexually antagonistic genes and Mishra *et al*.'s [78] list of genes with dominance reversed allele-specific expression between the sexes, suggesting the methods may complement one another. Similarly, Grieshop & Arnqvist [36] saw only a modest signal of additive sexually antagonistic effects using traditional quantitative genetic variance partitioning, but a definitive and strong signature of sex-reversed dominance ordination among strains using the same data. We propose that tests for dominance reversal – the signature of partially resolved antagonistic polymorphisms – paired with follow-up fitness validation tests would offer a powerful complementary approach to methods that aim to detect additive signals of antagonistic polymorphisms.

Besides those of the empirical examples above (table 1), three methods of testing for dominance reversals seem particularly promising: dominance ordination, allele-specific expression and allele-specific ATAC-Seq (assay for transposase-accessible chromatin with sequencing). While this list does not capture all potential approaches (e.g. [132,133]), we believe it represents some of the most promising newer methods. There are likely many existing datasets in the medical and agricultural literature that are ripe for reanalysis through the lens of dominance reversal using one or more of these methods.

Dominance ordination (explained in greater detail in electronic supplementary material S2) uses the ‘array covariances’ obtained from quantitative genetic data of a full diallel cross [115,119] to ordinate a panel of inbred strains from those whose fixed allelic variation (averaged across the genome) is mostly dominant over that of the other strains to those whose fixed alleles are mostly recessive to that of the other strains [36]. This process can be done separately for alternative contexts. The null expectation for underlying loci is that alternative alleles are either unconditionally dominant or unconditionally recessive, which would cause the strains to be in approximately the same order along the dominant-recessive continuum in both contexts. This null expectation would be detectable as a positive genetic correlation between contexts among the strain-specific array covariances ([36]; electronic supplementary material, figure S3.2C). By contrast, a negative cross-context genetic correlation among the array covariances would indicate that strains tend to be fixed for alleles that are dominant in one context but recessive in the other ([36]; electronic supplementary material, figure S3.2D), meaning that the underlying polymorphisms are dominance reversed. This method is currently best used as a quantitative genetic test of dominance reversal and does not reveal the causal polymorphisms underlying the signal. Possible avenues for further development of this method include extending it to other breeding designs and pedigree data [115] and/or applying the dominance ordination step at the level of chromosomes, linkage-blocks, or SNPs rather than strains or families.

By contrast, allele-specific expression analyses can identify specific genes with dominance reversed expression between alternative life stages, traits, tissues, sexes or environments. Sites with context-dependent allelic imbalance in expression represent candidate regions of the genome harbouring antagonistic polymorphisms, where further work would be required to identify candidate SNPs or indels as well as to validate their effects on fitness or fitness traits. We note that many allele-specific expression studies utilize crosses between compatible species because it increases the number of mRNA reads that can be confidently distinguished as having been inherited from one parent or the other (see below), but such data would not be relevant to the concept of dominance reversals resolving genetic conflicts (or maintaining genetic variation), as many of the variable sites would represent fixed differences between species. There are broadly two alternative allele-specific expression designs to identify dominance reversals: the ‘F_1_ hybrids’ approach and the ‘common reference’ approach [134]. Under both designs, transcriptomic reads from maternally and paternally inherited alleles can be identified and quantified by mapping F_1_ heterozygote reads back to both parental genotype-specific reference genomes [78,134]. Careful quality control and data filtering must be applied to rule out confounding parental effects and other sources of mapping bias (see [78]).

Once completed, relative read coverage between alternative alleles reveals allele-specific expression, and reversed allelic imbalance between contexts represents dominance reversed gene expression (electronic supplementary material, figure S2.6). Genes should only be considered as true positive reversals of allelic imbalance if the magnitude of allelic imbalance is significant in both contexts independently. A statistically significant allele-by-context interaction term could include genes that exhibit allelic imbalance in one context but not the other, which would indicate context-dependent dominance but not a dominance reversal, *per se*. This method could be enhanced by using long-read technologies such as PacBio, Oxford Nanopore or Iso-Seq [135–139] or linked-read sequencing applied to transcriptomic data [140] to better assign reads to the correct parental genome. In principle, single-cell technologies [137,141] could be integrated within the allele-specific expression methods to reveal the full atlas of genes that are dominance reversed across all tissue types.

ATAC-Seq could potentially reveal dominance reversed non-coding regions of the genome. ATAC-Seq identifies the genome-wide regions of organized DNA (i.e. chromatin) that are ‘open’ for transcription (i.e. euchromatin) by sequencing only the unwound euchromatin to build a database of active loci [142,143]. ATAC-Seq requires less tissue, time, troubleshooting and cost than CHIP-seq, while providing more information on the active loci, promoters and enhancers genome-wide. Since the euchromatin or heterochromatin state of DNA varies across contexts [144], allele-specific ATAC-Seq [145] could in principle reveal non-coding regions that are dominance reversed for transcriptional availability, including loci that may regulate dominance reversed allele-specific gene expression (such as the *cis*-regulatory binding sites in Box 1, S2). ATAC-Seq also reveals transcription factor binding site motifs [146] that could be integrated with the analysis of allele-specific expression data [145] to identify the regulatory networks that govern dominance reversed gene expression. These results could be assessed for the extent to which certain regulatory networks are predisposed to maintaining antagonistic polymorphisms via dominance reversal and could be evaluated across different classes of genetic trade-offs to assess the extent of overlap in regulatory sites and networks that are conducive to maintaining polymorphisms under different forms of antagonistic selection. Again, dominance reversed allele-specific expression and ATAC-Seq findings would have no functional or fitness basis on their own and should be interpreted with caution and/or validated by manipulative experimentation.

## 6. Conclusion

Beneficial reversals of dominance partially resolve genetic conflicts by improving population mean fitness, with the consequence of maintaining genetic variation. Studying whether different mechanisms compete or coordinate with dominance reversal to resolve genetic conflict has important implications for the evolution of genetic architecture and maintenance of genetic variation. Antagonistic genetic variation is likely to be enriched for dominance reversals because non-dominance reversed antagonistic polymorphisms are sensitive to being lost by drift or unequal strengths of selection between contexts. We suggest that dominance ordination, allele-specific expression and allele-specific ATAC-Seq represent promising methods of testing for these signatures of dominance reversal that are predicted to accompany antagonistic polymorphisms. There is ample scope for developing methods to identify signatures of dominance reversal and there are likely also many suitable datasets that already exist. We hope this article reignites research interest in dominance reversal as it bears broad and pivotal consequences to several areas of evolutionary biology.

## Data Availability

The data are available from Dryad Digital Repository: https://doi.org/10.5061/dryad.d7wm37q7k [147]. Supplementary material is available online [148]. The underlying Python code (for Box 1, electronic supplementary material S1) is available at https://gitfront.io/r/user-5416399/Ym11RGUhTp9L/simDominanceModifier/.
